# Dataset on renal tumor diameter assessment by multiple observers in normal-dose and low-dose CT

**DOI:** 10.1016/j.dib.2023.109672

**Published:** 2023-10-12

**Authors:** Jens Borgbjerg, Nis Elbrønd Larsen, Ivar Mjåland Salte, Niklas Revold Grønli, Elise Klæstrup, Anne Negård

**Affiliations:** a Department of Radiology, Akershus University Hospital, Sykehusveien 25, Nordbyhagen 1478, Norway; b Department of Radiology, Aarhus University Hospital, Aarhus, Denmark; c Institute of Clinical Medicine, University of Oslo, Oslo, Norway

**Keywords:** Renal tumor, Observer agreement, Tumor diameter, Computed tomography

## Abstract

Computed tomography-based active surveillance is increasingly used to manage small renal tumors, regardless of patient age. However, there is an unmet need for decreasing radiation exposure while maintaining the necessary accuracy and reproducibility in radiographic measurements, allowing for detecting even minor changes in renal mass size. In this article, we present supplementary data from a multiobserver investigation. We explored the accuracy and reproducibility of low-dose CT (75% dose reduction) compared to normal-dose CT in assessing maximum axial renal tumor diameter. Open-access CT datasets from the 2019 Kidney and Kidney Tumor Segmentation Challenge were used. A web-based platform for assessing observer performance was used by six radiologist observers to obtain and provide data on tumor diameters and accompanying viewing settings, in addition to key images of each measurement and an interactive module for exploring diameter measurements. These data can serve as a baseline and inform future studies investigating and validating lower-dose CT protocols for active surveillance of small renal masses.

Specifications Table

**Table:** 

Subject	Radiography and radiology
Specific subject area	Observer agreement data on computed tomography-based measurements of renal tumor diameters
Data format	Raw CSV PDF HTML
Type of data	Raw data files (CSV format files) Key images of measurements (PDF format file and interactive web-based module) Interactive web-based measurement explorer (website).
Data collection	We included CTs from the 2019 Kidney and Kidney Tumor Segmentation Challenge, in which patients underwent preoperative abdominal CT for a renal tumor. Using a web-based Digital Imaging and Communications in Medicine viewer, radiologist observers [residents (n = 2) and consultant (n = 4)] conducted blinded CT-based measurements of maximal axial diameter in 40 cases of renal tumors. These measurements were performed in both normal-dose CT and simulated 75% dose-reduced CT cases using a two-session mixed-order setup. Key images of each diameter measurement were obtained.
Data source location	Akershus University Hospital Oslo Norway
Data accessibility	Repository name: Mendeley data Data identification number: 10.17632/pf2bvmfsmb.1 Direct URL to data: https://data.mendeley.com/datasets/pf2bvmfsmb/5 Repository name: Github.com Direct URL to data (Interactive viewer): https://jmanden.github.io/imaging.github.io/LOAMViewerIndex.html 2019 Kidney and Kidney Tumor Segmentation Challenge [1] Repository name: Cancer imaging archive Direct URL to data: https://wiki.cancerimagingarchive.net/pages/viewpage.action?pageId=61081171
Related research article	Borgbjerg, J. *et al*. Radiation dose in CT-based active surveillance of small renal masses may be reduced by 75%: A retrospective exploratory multiobserver study. Research in Diagnostic and Interventional Imaging 5, 100019 (2023). doi:10.1016/j.redii.2022.100019

## 1 Value of the Data

• Assessment of absolute tumor size and growth in a reproducible manner is essential for safely carrying out active surveillance of renal masses. Data on the reproducibility of diameter measurements for this purpose are scarce - especially data obtained from using low-dose CT, and these initial data for normal- and low-dose CT can stimulate further research
• It is not currently clearly defined in the literature which characteristics (e.g., heterogeneity, contour, and attenuation) make renal tumors suitable for precise assessment as part of active surveillance. The presented data with key images and an interactive module to explore diameter measurements can benefit researchers in the priming of further studies.
• The dataset can be reused for sample size estimation in further studies in which investigators would like to obtain a prespecified certainty in the accuracy of observer agreement estimates in renal tumor diameter assessments. Furthermore, the data can be reused by other researchers to compare human versus computer precision in the development of automatic segmentation algorithms.

## 2 Data Description

### 2.1 Objective

CT-based active surveillance of renal masses has emerged as an increasingly utilized initial management strategy, even in younger patients [2]. There is an unmet need for investigating dose reduction strategies in serial imaging as part of CT-based active surveillance [3]. The data presented here are from a research letter titled “Radiation dose in CT-based active surveillance of small renal masses may be reduced by 75%. A retrospective exploratory multiobserver study“ [4], communicating a study undertaken to assess the agreement of renal tumor diameter measurements by different observers in low-dose CT and normal-dose CT.

### 2.2 Data

**Fig. 1:**
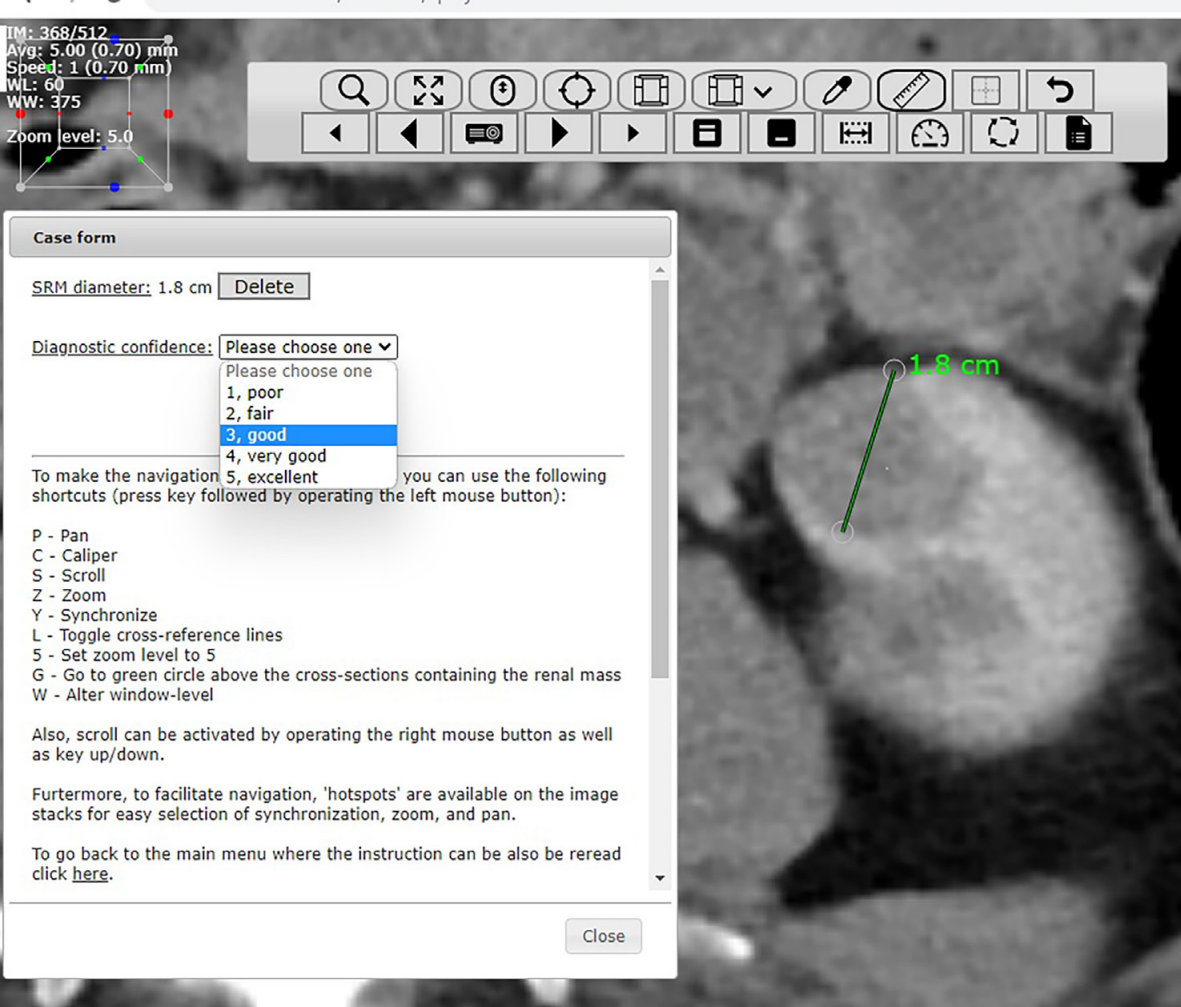
Representative image of the web-based DICOM viewer used by readers to assess maximal renal tumor diameter featuring a case report form.

**Fig. 2:**
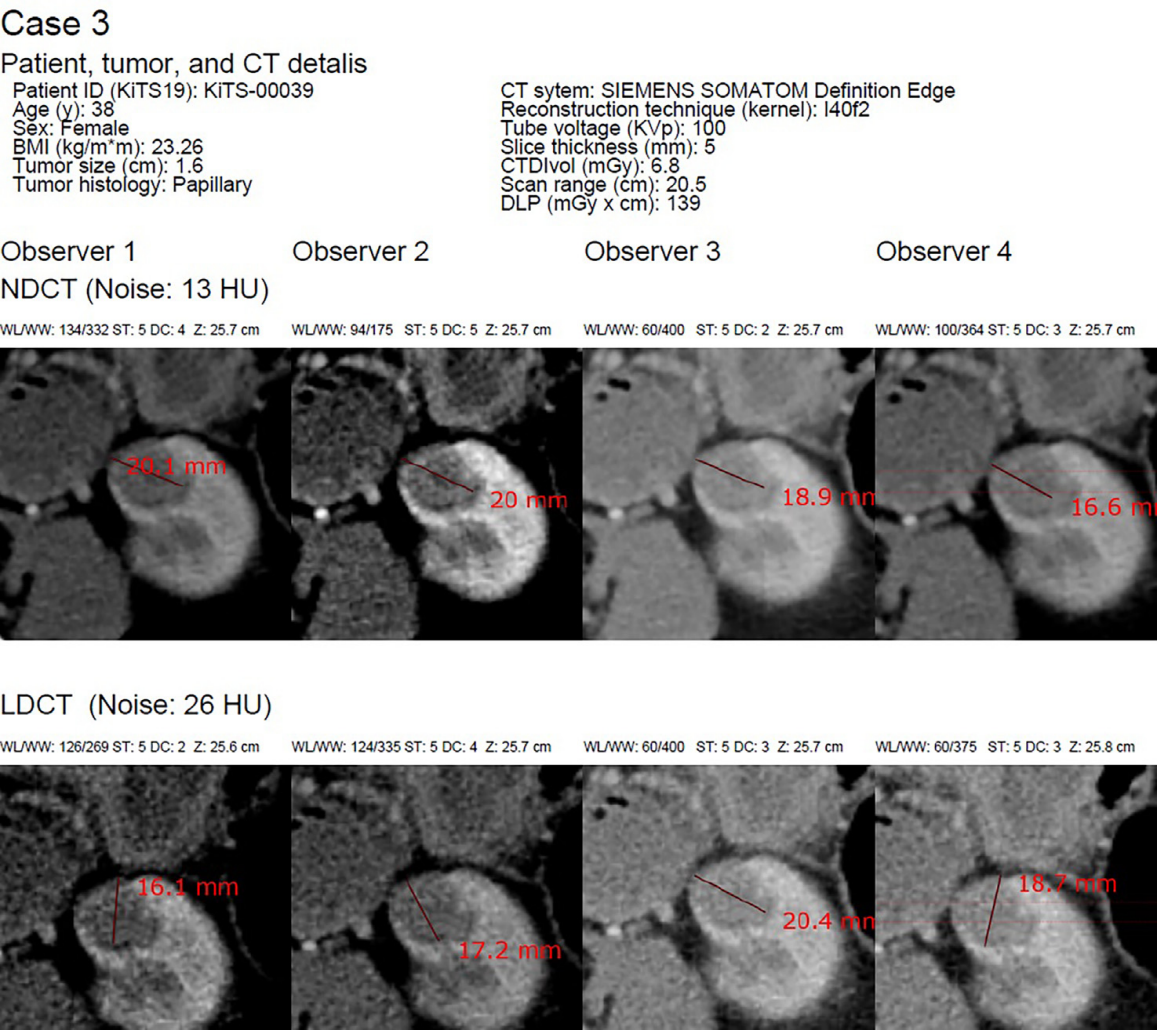
Subsection of the PDF imaging montage of Case 3.

The added material extends and complements the above-referenced research letter. The dataset includes two comma-separated files (CSV, “low-dose_6observers_40cases (1).csv” and “normal-dose_6observers_40cases (2).csv”) containing renal tumor diameters measured in millimeters in 40 CT cases as assessed by six radiologist observers for low-dose and normal-dose, respectively (Fig. 1). The CSV files have a column with the sequential case number and the identification number (i.e., *patient_id*) in relation to the 2019 Kidney and Kidney Tumor Segmentation Challenge dataset [1] as well as a column for each observer and a row for each tumor case. Furthermore, a PDF file includes a page for each tumor case containing an imaging montage of the 12 key images constituting tumor diameter assessments by the six observers in both low-dose and normal-dose CT (Fig. 2). Moreover, each page displays patient and CT characteristics (Table 1). In addition, for each individual diameter measurement, the parameters of chosen CT window level/width, slice thickness, diagnostic confidence in delineating the contour of the renal mass on a five-point scale, and craniocaudal level (z-level) of measurement by the observer in question are also shown in the PDF file.

BMI, body mass index; CTDIvol, CT dose index-volume; DC, Diagnostic confidence in delineating the contour of the renal mass on a five-point scale (1, poor; 2, fair; 3, good; 4, very good; and 5, excellent) as designated by the observer; DLP, dose length product; HU, Hounsfield unit; LDCT, low-dose CT; NDCT, normal-dose CT; Noise, the standard deviation in Hounsfield units obtained from a region of interest placed in the abdominal aortic lumen at the level of the renal tumor evaluated; RCC, renal cell carcinoma; SAFIRE, Sinogram Affirmed Iterative Reconstruction; ST, slice thickness as chosen by the observer; Slice thickness, original reconstructed slice thickness; Tumor size, as reported in the 2019 Kidney and Kidney Tumor Segmentation Challenge; WL/WW, CT window width and level as chosen by the observer.

**Table 1:** Demographics data, CT and Tumor Characteristics of study patients (n=40).

Patients	
Mean age (y)	58.9 (+/−14.8, 12–81)
Sex (female/male)	20 (50%)/20 (50%)
Mean BMI (kg/m^2^)	30.2 (+/−6.6, 16.2–45.2)
Tumor characteristics	
Mean tumor size (cm)	2.4 (+/−0.82, 1.2–4.5)
Tumor histology	
Clear cell RCC	26 (65%)
Papillary RCC	5 (13%)
Oncocytoma	4 (10%)
Chromophobe RCC	1 (3%)
Angiomyolipoma	2 (5%)
Multilocular cystic RCC	1 (3%)
Spindle cell neoplasm	1 (3%)
CT characteristics	
No. different CT systems	17
Image reconstruction	
Filtered back-projection	26 (65%)
SAFARI iterative reconstruction	14 (35%)
Mean scan length (cm)^a^	29.4 (+/−9.8, 20.0–60.0)
Mean tube voltage (kVp)	112.3 (+/−10.4, 1–5)
Mean tube current (mAs)	352.8 (+/−104.9, 169.8–558.6)
Mean slice thickness (mm)	3.8 (+/−1.4, 1–5)
Mean CTDI_vol_ (mGy)^b^	12.6 (+/−8.1, 4.5–39.2)
Mean DLP (mGy × cm)^b^	342.1 (+/−188.4, 121.9–917.3)
Mean effective dose (mSv)^b^^c^	5.1 (+/−2.8, 1.8–13.8)
Mean noise (NDCT, HU)^d^	17.1 (+/− 6.3, 8–32)
Mean noise (LDCT, HU)^d^	34.2 (+/− 12.6, 16–64)

Unless otherwise indicated, data are numbers and data in parentheses are percentages.

Mean data are presented with standard deviation and range in parentheses.

a From the diaphragm to the abdominal aortic bifurcation.

b Data available for 22 out of 40 CT scans.

c Effective dose = DLP × abdominal weighting factor (=0.015 mSv × mGy^−1^ × cm^−1^).

d Obtained from a region of interest placed in the abdominal aortic lumen at the level of the renal tumor evaluated. BMI, body mass index; RCC, renal cell carcinoma; DLP, dose length product; CTDIvol, CT dose index-volume; HU, Hounsfield unit; LDCT, low-dose CT; NDCT, normal-dose CT; SAFIRE, Sinogram Affirmed Iterative Reconstruction.

### 2.3 Online Interactive Interface for Data Exploration

**Fig. 3:**
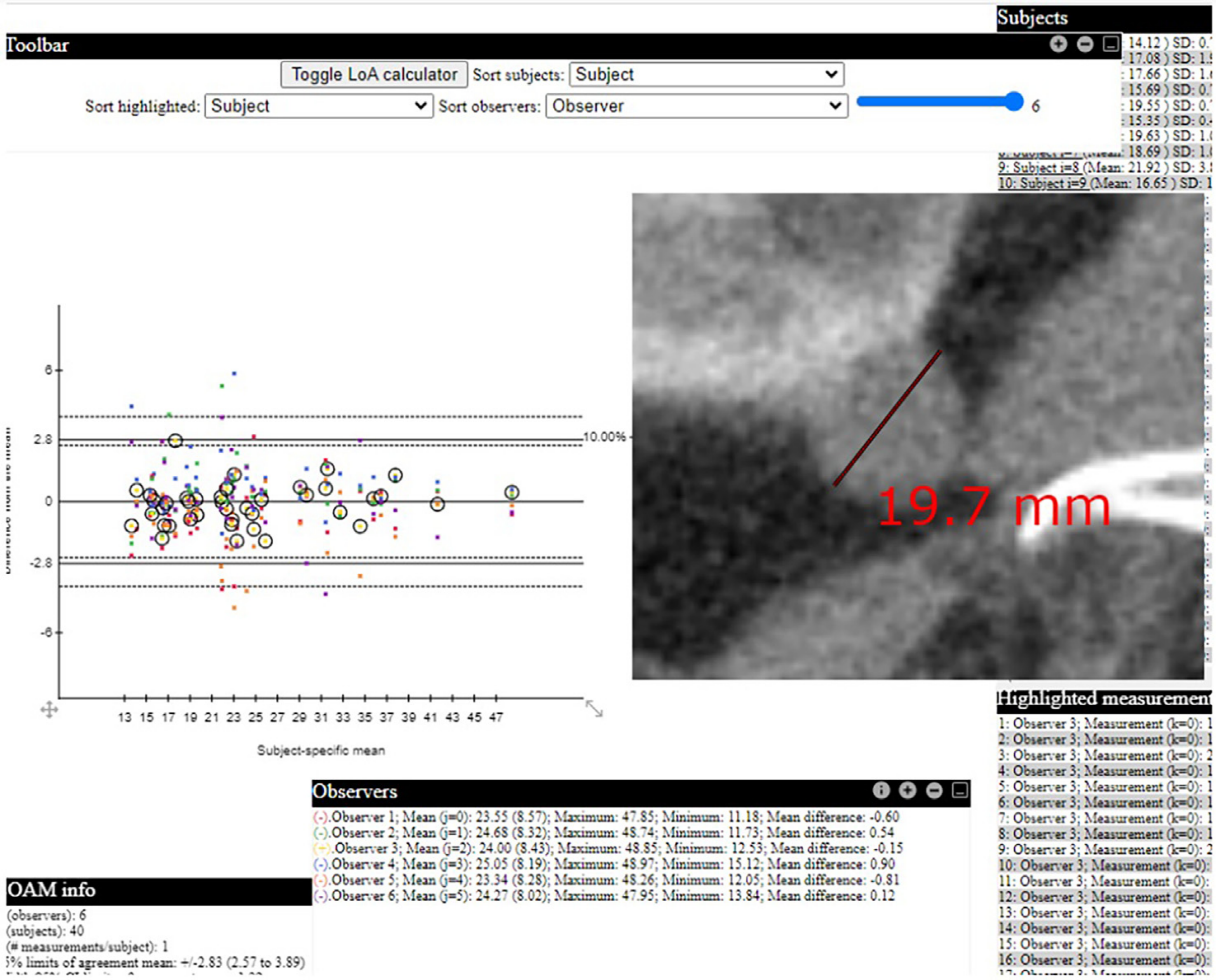
The interactive web-based statistical module with an agreement plot based on the low-dose CT measurements by six observers in 40 cases. Measurements of the 6 observers are shown in the agreement plot. The measurements by observer 3 have been highlighted in the plot, and a key image of the measurement on CT renal tumor case 7 by that observer is displayed.

We developed a statistical module to view the dataset with a user-friendly interactive graphical user interface written in JavaScript as a single-page HTML web application available at https://jmanden.github.io/imaging.github.io/LOAMViewerIndex.html. The module processes the diameter measurements of the aforementioned CSV files by utilizing the statistical method of the Limits of agreement with the mean (LOAM) as formulated by Christensen et al. [5]. The user interface has two tables with characteristics of the observers and subjects (tumors). In addition, an interactive agreement plot is shown; the observed differences of the individual measurements are plotted against the observed subject-specific average, and this plot is equipped with horizontal lines representing the 95% LOAM (Fig. 3). Measurement points on the agreement plot are coupled to the corresponding key images saved as JPEG files enabling a swift display of the caliper placements. On mouse over functionality will highlight measurements in the “Highlighted measurements” table from where a key image can be shown. Hence, this module enables the exploration of measurement outliers and, compared to a static plot, can more clearly delineate measurements by a given observer (Fig. 3). Another functionality is to sort the “Subjects” table according to the greatest measurement deviations where these can be highlighted and visualized through the “Highlighted measurements.” An “Information icon” is available at the top row of each table to facilitate usage.

## 3 Experimental Design, Materials and Methods

### 3.1 Patients and CT Datasets

Experimental design has been described briefly elsewhere [4]. In more detail, we used CT scans depicting a renal mass in the late-arterial contrast phase from the 2019 Kidney and Kidney Tumor Segmentation Challenge (KiTS19) dataset [1]. Imaging was performed during routine care of patients undergoing partial or radical nephrectomy at the University of Minnesota Medical Center. Many CT scans were performed at referring institutions and are heterogeneous regarding scanner manufacturers and protocols. The study population consisted of 40 patients. Initial eligibility criteria were as follows: 1) tumor size <= 4 cm (consistent with the definition of a small renal mass), 2) intravenous contrast enhancement in the late arterial phase with sufficient enhancement of the renal cortex to allow straightforward differentiation between the cortex and the medulla [6], 3) and non-infiltrating tumor as determined by an abdominal radiologist with 11 years of CT experience. These criteria identified a total of 38 patients from the KiTS19 dataset.

To obtain a total sample of 40 patients comparable to that included in a previous study [7], two additional randomly selected patients who met the above criteria, except for the size category >4 and <= 5 cm, were also included. We used a previously demonstrated technique for simulating lower-dose CT scans by adding noise to DICOM images, corresponding to a 75% dose reduction [8].

### 3.2 Readers

Two months after the previously described patient selection process was completed, the normal-dose and low-dose CT datasets were independently reviewed by six readers from Akershus University Hospital and Aarhus University Hospital. Participating readers were recruited by e-mail invitation. These readers comprised four fellowship-trained abdominal radiologists with 30, 11, 8, and 6 years of CT experience and two radiology residents with 5 and 2 years of CT experience.

### 3.3 Case Assessment Platform and Measurement

A web-based platform for facilitating observer performance studies in imaging research described in a prior research study was customized and used for the investigation at hand [9]. This platform uses PHP and MySQL for web interfaces and a database, respectively, and provides an environment where image readings can be conducted using an internet browser. The platform was coupled with a GPU (graphics processing unit)-accelerated web-based Digital Imaging and Communications in Medicine (DICOM) viewer based on JavaScript and WebGL 2.0 with multiplanar reconstruction capability [10] and was equipped with a case report form (Fig. 1). The CT scans were randomized and transferred onto the platform database. Readers were provided with written case assessment instructions and videos demonstrating the web-based DICOM viewer functionality with examples of caliper placement (Supplementary video file A). Readers practiced on sample CT scans to become familiar with caliper placement and the DICOM viewer interface. A two-session mixed-order setup was employed with at least a 10-day washout interval between case assessment sessions. The DICOM viewer presented orthogonal multiplanar reconstruction to facilitate the localization and measurement of the maximum axial diameter, with windowing and leveling at the discretion of each observer. Observers were blinded to all technical parameters, clinical data, previous measurements, key images, and measurements taken by other observers. A key image of each caliper placement was automatically saved in the database, as well as the *x*–*y*–*z* coordinates of each two-point caliper placement. In addition, for each case, observers rated diagnostic confidence in delineating the contour of the renal mass on a five-point Likert scale (1, poor; 2, fair; 3, good; 4, very good; and 5, excellent). Furthermore, the radiologist who also determined case eligibility placed a region of interest in the abdominal aorta at the level of the renal tumor in each scan to obtain the image noise (i.e., the standard deviation of the region of interest Hounsfield units). The JavaScript module jsPDF (https://github.com/parallax/jsPDF) was coupled with the measurement data stored in the MySQL database to automatically generate the PDF image montage.

## Limitations

None.

## Ethics Statements

The Institutional Review Board approved the data collection, with informed consent obtained from participating radiologists (CrudeNoise_SRM_LDCTvsNDCT_Protocol_23.08.21). All procedures followed were in accordance with institutional guidelines and the Declaration of Helsinki.

## CRediT authorship contribution statement

**Jens Borgbjerg:** Data curation, Methodology, Software, Supervision, Writing – original draft. **Nis Elbrønd Larsen:** Data curation, Writing – review & editing. **Ivar Mjåland Salte:** Data curation, Visualization, Writing – review & editing. **Niklas Revold Grønli:** Data curation, Writing – review & editing. **Elise Klæstrup:** Data curation, Writing – review & editing. **Anne Negård:** Conceptualization, Data curation, Writing – review & editing.

## Data Availability

Data of ‘Radiation dose in CT-based active surveillance of small renal masses may be reduced by 75%. A retrospective exploratory multiobserver study’ (Original data) (Mendeley Data).

## Appendix Supplementary materials

Image, video 1
